# Climbing Fiber Regulation of Spontaneous Purkinje Cell Activity and Cerebellum-Dependent Blink Responses^1,2,3^

**DOI:** 10.1523/ENEURO.0067-15.2015

**Published:** 2016-01-25

**Authors:** Riccardo Zucca, Anders Rasmussen, Fredrik Bengtsson

**Affiliations:** 1 Synthetic Perceptive Emotive and Cognitive Systems Laboratory, Center for Autonomous Systems and Neuro-robotics, Information and Communication Technologies Department, Universitat Pompeu Fabra, 08002 Barcelona, Spain; 2 Department of Neuroscience, Erasmus Medical Center, 3000 DR, Rotterdam, The Netherlands; 3 Department of Experimental Medical Science, Section for Neurophysiology, Lund University, SE-221 84 Lund, Sweden

**Keywords:** climbing fibers, eyeblink conditioning, Purkinje cell, simple spikes, spontaneous background firing

## Abstract

It has been known for a long time that GABAergic Purkinje cells in the cerebellar cortex, as well as their target neurons in the cerebellar nuclei, are spontaneously active. The cerebellar output will, therefore, depend on how input is integrated into this spontaneous activity. It has been shown that input from climbing fibers originating in the inferior olive controls the spontaneous activity in Purkinje cells. While blocking climbing fiber input to the Purkinje cells causes a dramatic increase in the firing rate, increased climbing fiber activity results in reduced Purkinje cell activity. However, the exact calibration of this regulation has not been examined systematically. Here we examine the relation between climbing fiber stimulation frequency and Purkinje cell activity in unanesthetized decerebrated ferrets. The results revealed a gradual suppression of Purkinje cell activity, starting at climbing fiber stimulation frequencies as low as 0.5 Hz. At 4 Hz, Purkinje cells were completely silenced. This effect lasted an average of 2 min after the stimulation rate was reduced to a lower level. We also examined the effect of sustained climbing fiber stimulation on overt behavior. Specifically, we analyzed conditioned blink responses, which are known to be dependent on the cerebellum, while stimulating the climbing fibers at different frequencies. In accordance with the neurophysiological data, the conditioned blink responses were suppressed at stimulation frequencies of ≥4 Hz.

## Significance Statement

The cerebellum is vital for many important functions, including predicting sensory events, adjusting reflexes, allowing smooth movements, and acquiring associations between different stimuli. Purkinje cells, in the cerebellar cortex, have a spontaneous activity that is regulated by climbing fiber input from the inferior olive. Here we show that stimulating climbing fibers result in a frequency-dependent suppression of Purkinje cell activity. Moreover, such stimulation abolishes the expression of conditioned blink responses, which are known to rely on the cerebellum. These results demonstrate that cerebellar function is crucially dependent on normal levels of spontaneous activity in Purkinje cells.

## Introduction

Purkinje cells of the cerebellar cortex receive input through two afferent pathways. The mossy/parallel fiber pathway carries input from a large number of nuclei in the brainstem and the spinal cord, while the climbing fiber pathway carries input from the inferior olive. Whereas activation of the mossy/parallel fibers elicits simple spikes in Purkinje cells, activating climbing fibers results in the generation of complex spikes (Eccles et al., 1967). Simple spikes consist of single Na^+^-dependent action potentials. At rest, Purkinje cells fire at an average rate of ∼44 Hz (Armstrong and Rawson, 1979) but can be modulated up to 200 Hz (Thach, 1967). Complex spikes, in comparison, occur at low rates, normally between 0.5 and 1.5 Hz, and rarely exceed 5 Hz (Armstrong and Rawson, 1979; Womack and Khodakhah, 2002; Bengtsson et al., 2004; Cerminara and Rawson, 2004). While input from mossy/parallel fibers and climbing fibers can modulate their activity, Purkinje cells are tonically active (Woodward et al., 1974; Gähwiler, 1975; Llinás and Sugimori, 1980; Hounsgaard and Midtgaard, 1988; Häusser and Clark, 1997; Raman and Bean, 1999; Cerminara and Rawson, 2004). The direct effect of climbing fiber input to Purkinje cells is depolarization. However, increasing or decreasing the climbing fiber firing frequency beyond its normal range results in decreased or increased simple spike firing, respectively (Colin et al., 1980; Rawson and Tilokskulchai, 1981; Montarolo et al., 1982; Demer et al., 1985; Andersson and Hesslow, 1987b; Bengtsson et al., 2004; Cerminara and Rawson, 2004).

Several lines of evidence suggest that balanced simple and complex spike activity is essential for normal behavior (Llinás et al., 1975; Zbarska et al., 2008). Consequently, lesions of the inferior olive, as well as blocking the effect of GABA in the cerebellar nuclei, which results in the inhibition of the olive, abolishes cerebellum-dependent conditioned responses (Yeo et al., 1986; Parker et al., 2009). Balanced output from the inferior olive relies on GABAergic input from the cerebellar nuclei, which in turn relies on activity in Purkinje cells (Andersson and Hesslow, 1987a,b; Lang et al., 1996; Bengtsson et al., 2004; Bengtsson and Hesslow, 2006, 2013; Bazzigaluppi et al., 2012; Chaumont et al., 2013; Najac and Raman, 2015). Although this interdependence between different parts of the cerebellar circuit is a fundamental principle of cerebellar function, the exact relationship between climbing fiber activity and simple spike activity has not yet been fully described. In this study, we provide a detailed analysis of the effect of climbing fiber activation at different frequencies on simple spike activity and the expression of cerebellum-dependent conditioned blink responses.

## Materials and Methods

### Surgery

Eight male ferrets were initially anesthetized with a mixture of O_2_ and air, with 1.5–2% isoflurane (Baxter Medical), which was subsequently replaced intravenously by propofol (10 mg/ml Diprivan; AstraZeneca). During anesthesia, a tracheotomy was performed, and the gas was led directly into a tracheal tube. The end-expiratory CO_2_ concentration, arterial blood pressure, and rectal temperature were monitored and kept within physiological limits throughout the experiment. During the whole experiment, infusion was given intravenously [50 mg/ml glucose and isotonic acetate Ringer’s solution (proportion, 1:1) with 0.004 mg/ml albumin fraction V (from bovine serum; Sigma-Aldrich), 2 mg/kg/h]. After fixation of the head in a stereotaxic frame, the skull was opened on the left side, and the caudal half of the left cerebral hemisphere, together with a substantial part of the thalamus on the left side, were removed by aspiration. The animals were decerebrated by sectioning the brainstem with a spatula 1–2 mm rostral to the superior colliculus. After decerebration, anesthesia was discontinued. With the cerebellum and colliculi exposed, a pool was constructed of cotton-reinforced agar and filled with warm high-density perfluorocarbon liquid (FC-40 Fluorinert; 3M). To ensure mechanical stability, animals were immobilized by curare, artificially ventilated, and kept hanging by the spine. A bilateral pneumothorax was performed to minimize chest movements and movements caused by changes in venous blood pressure. The dura covering the cerebellum was removed, and the surface was covered with agarose gel (15 mg/ml) to improve stability and prevent edema. This study was reviewed and approved by the local Swedish Ethical Committee.

### Climbing fiber stimulation

**Figure 1.**
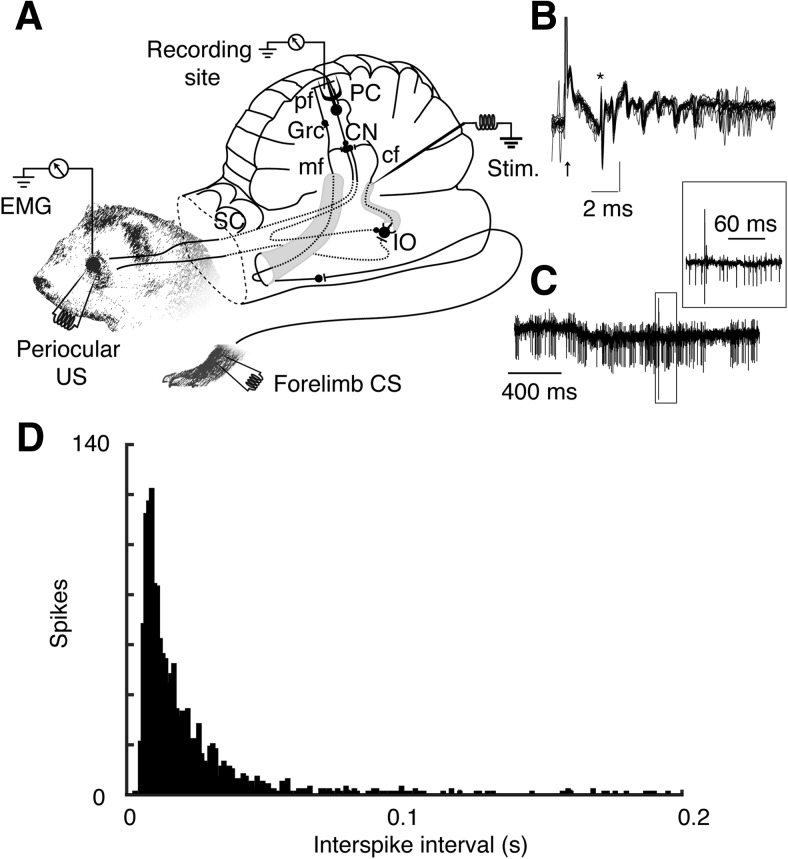
Experimental setup, cerebellar circuit diagram, and single unit records. ***A***, Schematic illustration of the experimental setup with recording and stimulation sites. mf, Mossy fiber; cf, climbing fiber; pf, parallel fiber; PC, Purkinje cell; GrC, granule cell; CN, cerebellar nuclei; IO, inferior olive; SC, superior colliculus. ***B***, Complex spikes evoked by climbing fiber stimulation. Thin black traces represent ten superimposed complex spikes. Thick trace is the average waveform. The asterisk indicates the onset of the complex spike and the black arrow indicates the time of the stimulation. ***C***, Extracellular recording of a representative Purkinje cell. Bottom trace shows an unfiltered recording and the inset shows, in greater detail, the complex spike evoked by climbing fiber stimulation. ***D***, Archetypal interspike interval distribution before the start of the climbing fiber stimulation.

The experimental setup, including stimulation and recording sites, is illustrated in Figure 1*A*. Climbing fibers were stimulated in the ipsilateral inferior cerebellar peduncle by lowering a tungsten electrode (diameter, 30 μm; uninsulated tip, 50 μm) 4.0–5.0 mm below the posterior cerebellar surface, at an angle of 45°, 4 mm lateral to the midline, and 4 mm rostral to the caudal border of the vermis. While tracking, single stimulus pulses were applied and the evoked field potentials were recorded in the C3 zone of the hemispheric lobule VI, which was identified by previously established criteria (Hesslow, 1994a; Hesslow and Ivarsson, 1994). The effectiveness of all stimulation sites and stimulus intensities were verified again and adjusted, if necessary, when recording the activity of single Purkinje cells.

### Purkinje cell recording

Five extracellular single-unit records of Purkinje cells were obtained using microelectrodes with pulled and ground tips, 30–40 μm metal core diameter (Thomas Recording). When a Purkinje cell with the appropriate input characteristics was found, we began stimulating climbing fibers at 0.5 Hz after which the frequency was increased by 0.5 Hz every 5 min up to 4 Hz. Above this frequency, the stimulation was increased by 1 Hz every 5 min. In one experiment, we included 60 s periods between every switch in stimulation frequency during which no stimulation was given. This was done to determine whether the effect on background activity lasted beyond the stimulation. To optimize the recording stability, animals were curarized when we searched for or recorded the activity of Purkinje cells.

### Analysis of Purkinje cell activity

Voltage changes related to neural activity were passed unfiltered through an amplifier and sampled at 40 kHz using a Power 1401 A/D converter unit (CED). Action potentials were isolated on-line with Spike2 software (version 7.11; CED Electronics Design). All recordings were subsequently reanalyzed off-line. Identification of complex spikes followed a series of inclusion criteria: the presence of spikelets after the large initial spike (Fig. 1*B*), a short latency inhibition ∼10 ms after the complex spike (Fig. 1*C*), as well as archetypal interspike interval distributions (Fig. 1*D*). As the firing rate of Purkinje cells is highly variable between different cells, firing rates were normalized relative to a baseline segment recorded before the start of the stimulation protocol. Population responses were calculated by averaging over Purkinje cells for the same session. Unless otherwise stated, we report activity as the mean ± SEM.

### Eyeblink conditioning

Three ferrets were trained in a standard delay conditioning paradigm, where the conditional stimulus (CS) was a 320 ms, 50 Hz train of 1 ms electrical pulses, applied subcutaneously through a pair of needle electrodes inserted ∼5 mm apart through the skin of the left forelimb (intensity range, 1.2–2 mA). Prior to training, we verified that the stimulation did not elicit any eye muscle activity. The unconditional stimulus consisted of three 1 ms pulses at 50 Hz, with a stimulation intensity of 3 mA, applied bilaterally to the periocular skin, 300 ms after the onset of the conditional stimulus. We used a pseudo-random 16 ± 1 s intertrial interval. The stimuli used in this study are known to be effective for eyeblink conditioning in this preparation (Hesslow and Ivarsson, 1996; Ivarsson et al., 1997). Conditioning started approximately 1 h after completion of the surgery and continued until the animal emitted conditioned eyeblink responses (ECRs) on at least 7 of 10 consecutive CS-alone trials. During training, the animals were paralyzed through curare. This paralysis was interrupted every 30 min to monitor the behavioral response.

### Behavioral analysis

To examine the acquisition of conditioned blink responses we recorded electromyographic (EMG) activity bilaterally through pairs of stainless steel electrodes. The signal was amplified, high-pass filtered at 5 kHz, and digitized at 40 kHz with a Power 1401 data acquisition analog-to-digital (A/D) converter unit (CED). Data analysis was performed using Spike2 version 7 software (CED) and custom routines developed in Matlab (MathWorks). Eyeblink responses from each valid trial were examined off-line. The recorded EMG was rectified, and the mean power of the signal was obtained calculating the root mean square in a 5 ms time window. In general, ECRs could easily be discriminated by visual inspection of the raw signal. Muscle activity between 50 and 298 ms after CS onset, with at least twice the amplitude of spontaneous muscle activity 0-100 ms before CS, was considered to be an ECR. The onset latency was defined as the time when activity exceeded and stayed above this threshold for at least 50 ms. Responses with a latency of <50 ms were classified as alpha responses and were excluded from the valid ECRs trials. Means of ECR incidence (i.e., the ratio between trials that elicited an ECR and the total number of trials within a block) and baseline activity were calculated and plotted for blocks of 10 consecutive trials. Onset latency, peak latency, and response amplitude were determined only for those trials with an ECR. All group data are presented as the mean ± SEM.

## Results

### Purkinje cell recordings

**Figure 2.**
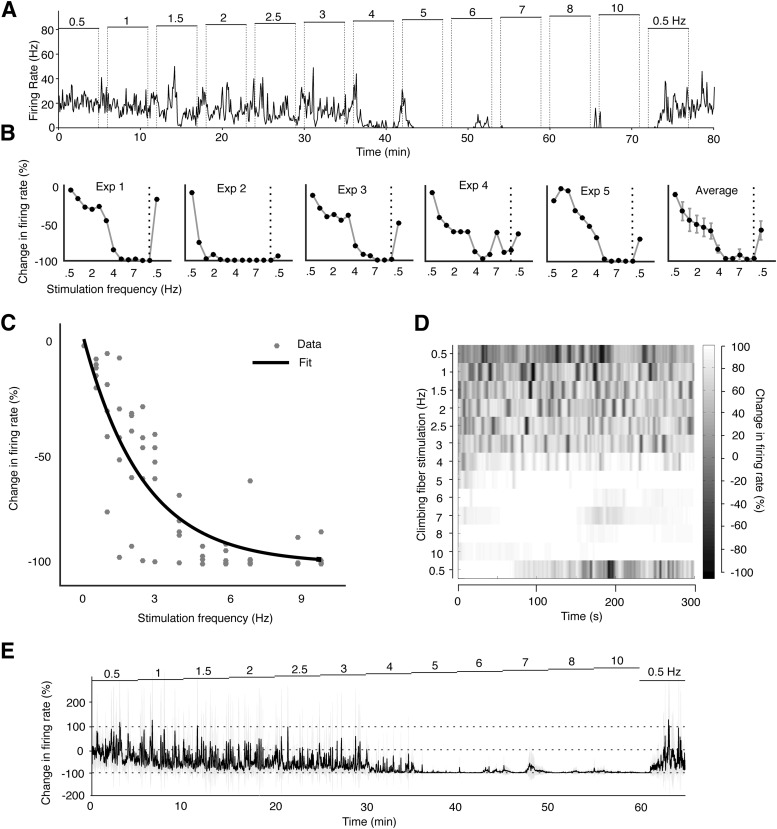
Stimulating climbing fibers suppresses Purkinje cell activity in a frequency dependent manner. ***A***, Time course of the simple spike suppression in a Purkinje cell during a complete experimental session. Climbing fibers were stimulated with electrical pulses (duration, 0.1 ms; intensity, 240 μA), for 5 min at incremental frequencies, with 60 s breaks in between each switch in frequency. The firing rate was estimated through convoluting the spike train with a Gaussian kernel (sigma, 2 s). ***B***, Change in Purkinje cell activity as a function of the climbing fiber stimulation frequency in each of the five cells, as well as the average change (right). ***C***, Scatterplot illustrating the relation between the climbing fiber stimulation frequency on the *x*-axis and changes in Purkinje cell activity on the *y*-axis. The data fits an exponential curve (black line) described by a coefficient of 0.397. The best fit was obtained through the Matlab Curve fitting toolbox (MathWorks). ***D***, Average raster plot of simple spike firing rate changes over time. The color of the shadings indicates simple spike activity changes relative to the pre-stimulation baseline (baseline = 0%). Lighter areas indicate inhibition of simple spike firing (i.e., 100% equals a complete suppression) and darker areas indicate increased activity. ***E***, Time course of the simple spike activity changes over the entire stimulation session averaged for the five experiments. Each data point corresponds to the firing rate change over a 100 ms window. Light gray shadings represent a 95% confidence interval.

We recorded the activity of Purkinje cells in the blink-controlling C3 zone of the cerebellar cortex, identified by characteristic field potentials evoked by periocular stimulation (Bengtsson et al., 2004; Jirenhed et al., 2007; Jirenhed and Hesslow, 2015). Five Purkinje cells with periocular and climbing fiber input were recorded from five different animals. The recordings were stable, with a good signal-to-noise ratio (Fig. 1*C*). The Purkinje cell firing rate before the start of the stimulation was between 8.85 and 33.7 Hz (mean, 20.2 ± 9.37 Hz). Climbing fibers were stimulated in a stepwise incremental fashion starting at 0.5 Hz and increasing 0.5-1 Hz every 5 min. The exact frequencies used are shown in Figure 2*A*. A complete experimental session lasted for 65 min (78 min when the 60 s interval between switches in frequency was introduced). At all frequencies, the stimulation reliably elicited a complex spike with the expected 2-3 ms latency (Fig. 1*B*). Sometimes, the stimulation also elicited a second complex spike that was indirectly evoked by the climbing fiber reflex (Eccles et al., 1966). However, in these experiments, the stimulation strength was adjusted to minimize the occurrence of such reflexive climbing fiber responses.

### Effect of climbing fiber stimulation on Purkinje cell firing rate

**Figure 3.**
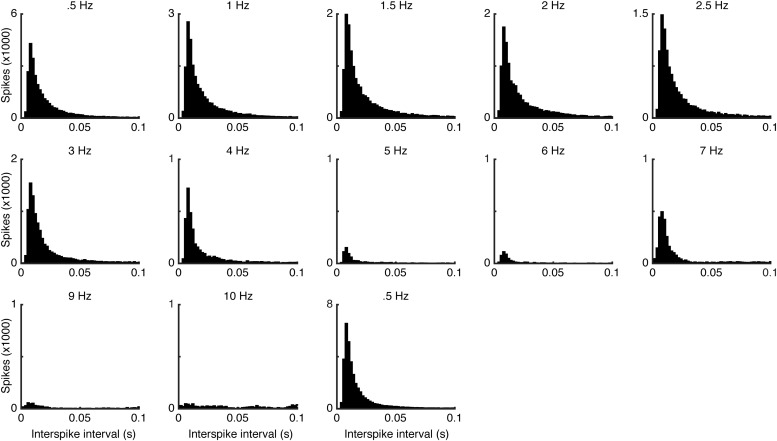
Interspike interval distributions for each climbing fiber stimulation frequency (bin size, 2 ms).

A repeated measures ANOVA with a Greenhouse–Geisser correction showed that increasing the frequency of the climbing fiber stimulation had a reliable inhibitory effect on Purkinje cell firing (*F*_(1713,6850)_ = 21.737, *p* = 0.0012; Table 1). An example of the effect of sustained climbing fiber activation over an entire experimental session is illustrated in Figure 2*A*. Whereas stimulation with intensities below the threshold for eliciting complex spikes did not induce any suppression at any frequency tested, above threshold stimulation in the range 0.5-10 Hz, suppressed simple spike activity in a graded manner (Fig. 2*B–E*). *Post hoc* testing, using Bonferroni correction, revealed that stimulation at 4 Hz consistently resulted in a strong suppression of Purkinje cell activity (84.04 ± 5%, *p* = 0.0067), and stimulation at 5 Hz all but silenced the Purkinje cells (96.6 ± 1.5%, *p* = 0.00002). As illustrated in Figure 3, increasing the climbing fiber stimulation frequency, and thus decreasing Purkinje cell activity, did not affect the interspike interval distribution substantially. This inhibitory effect is consistent with results reported in previous investigations in which a complete suppression was induced at stimulation frequencies of 4–5 Hz (Rawson and Tilokskulchai, 1981; Demer et al., 1985). In one Purkinje cell, a 76.3% reduction in simple spike firing was observed already at 1 Hz, and increasing the stimulation frequency to 1.5 Hz all but silenced the cell (97.19% suppression over the 5 min session).

**Table 1:** Statistical analysis

	Data structure	Type of test	Power
a.	Normal	Repeated-measures ANOVA	0.0012
	4 Hz stimulation	*Post hoc* (Bonferroni corrected)	0.0067
	5 Hz stimulation	*Post hoc* (Bonferroni corrected)	0.00002
	0.5 Hz stimulation (final session)	*Post hoc* (Bonferroni corrected)	0.812

In the last stimulation session, we switched back to a stimulation frequency of 0.5 Hz. This resulted in a recovery of the Purkinje cell activity, yet the suppression persisted for an average of 118.6 ± 42.56 s. The average firing rate in the last 0.5 Hz stimulation session was 59.3 ± 12.5% (*p* = 0.812), although the Purkinje cell that exhibited almost complete suppression at 1.5 Hz remained suppressed throughout this session (Fig. 2*B*, Exp 4).

### Contribution of post-complex spike pauses to the simple spike suppression

Complex spikes are usually followed by a characteristic pause in Purkinje cell activity that can last from 10 ms to a couple of hundred milliseconds (Latham and Paul, 1971; Simpson et al., 1996). Could the suppression of Purkinje cell activity reflect the combined effect of all post-complex spike pauses? To quantify the contribution from the post-complex spike pauses to the simple spike suppression, we calculated the average duration of the post-complex spike pause (population mean, 0.038 ± 0.021 s). Then we estimated the firing rate that should have been observed only if these post-complex spike pauses contributed to the suppression. The firing rate variation relative to the baseline was then recalculated. The estimated contribution of the post-complex spike pauses to the total suppression would account for at most 1.9% (range, 1.0–3.5%) at the lowest stimulation frequency used (0.5 Hz), and 34% (range, 18–63), at the highest stimulation frequency used (10 Hz).

### Effects of sustained climbing fiber discharge on overt behavior

After stable conditioning had been achieved, curarization was discontinued, and the animals were tested with the climbing fiber stimulation protocol. This protocol consisted of seven experimental sessions separated by 5 min resting periods in which no stimulation was applied. Each session was composed of four blocks of 10 trials. The first block, which consisted of 10 paired trials, served as a control condition. Following this were two blocks, each consisting of 10 CS-alone trials. During these CS-alone trials, climbing fibers in the cerebellar peduncle were stimulated at a fixed frequency. Between sessions, we increased the frequency of the climbing fiber stimulation in a stepwise manner going from 1 to 4.5 Hz, in 0.5 Hz increments. To avoid the presence of stimulation artifacts in the electromyographic trace, climbing fiber stimulation was stopped for 1 s, starting 200 ms before the CS onset. A final block without climbing fiber stimulation served as a second control condition.

**Figure 4.**
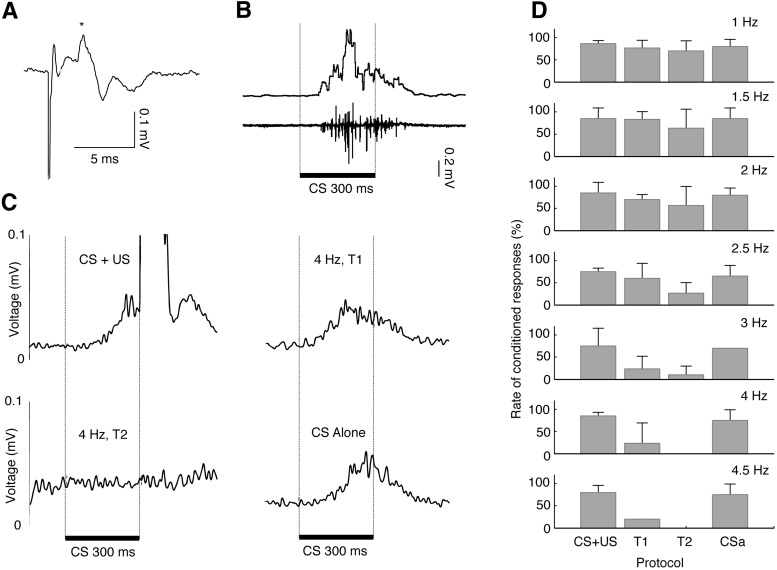
***A***, Average of 10 consecutive field potentials recorded on the cerebellar cortex following direct stimulation of climbing fibers. Asterisk indicates the climbing fiber response. ***B***, EMG from the orbicularis oculi muscle recording on a single CS-alone trial in a trained animal. The top trace shows a rectified and smoothed trace (smoothing window 10 ms). The bottom trace shows the raw signal. ***C***, Rectified and smoothed EMG on paired trials without climbing fiber stimulation (top left), CS-alone trials with climbing fiber stimulation at 4 Hz (top right and bottom left), and on CS-alone trials without climbing fiber stimulation (bottom right). ***D***, Effect of sustained climbing fiber stimulation at 1–4.5 Hz on the rate of conditioned eyeblink responses (ECR). Each bar plot shows the percentage of ECRs (+ SEM), in blocks of 10 trials, for each stimulation frequency.

**Figure 5.**
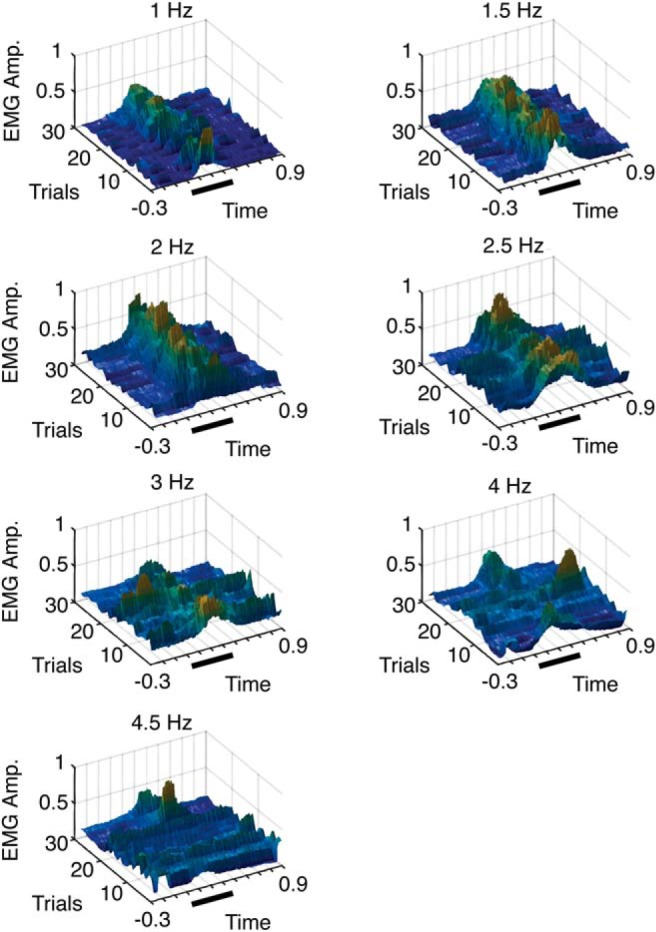
3D surface plots illustrating orbicularis oculi EMG in one animal in the last three test blocks for each stimulation frequency. Data were rectified and binned over a 50 ms window and normalized to the highest value over the seven recording sessions.

All animals developed ECRs at a normal rate, reaching a stable level of conditioning within 3–5 h (∼500–700 trials). At this point, the CS elicited ECRs on 86 ± 5.7% of the trials. Figure 4 illustrates a climbing fiber field potential recorded on the cerebellar cortex (Fig. 4*A*) and an overt ECR recorded with EMG electrodes (Fig. 4*B*). The mean onset latency of the ECRs was 163 ± 39 ms, and the mean peak latency was 236 ± 44 ms after CS onset. The acquisition process was similar to that observed in previous studies (Hesslow et al., 1999; Wetmore et al., 2014). Mirroring the effect on Purkinje cell activity, climbing fiber stimulation suppressed ECRs in a frequency-dependent manner (Figs. 4*C*-*D*, 5). The rate of ECRs in the paired trials preceding the stimulation did not differ between sessions (mean ECRs, 81.6 ± 5%), and it was comparable to the ECR frequency observed at the end of acquisition, indicating that no extinction occurred during or between the experimental sessions. Stimulating the climbing fibers at frequencies from 1 to 2.5 Hz did not result in a substantial reduction of ECRs. However, stimulation at ≥3 Hz induced a strong suppression of ECRs. For the highest stimulation intensities tested (4–4.5 Hz), ECR expression was completely blocked in the second experimental block (Figs. 4*C*-*D*, 5). For all the stimulation frequencies tested, the rate of ECRs in the fourth block, when climbing fiber stimulation was discontinued, returned to the control level (mean, 76 ± 6.7%).

Consistent with the fact that Purkinje cells inhibit the cerebellar nuclei, climbing fiber stimulation, which induces a suppression of Purkinje cell activity, resulted in increased EMG activity at the higher stimulation frequencies (Fig. 5). On a few occasions, we also observed muscle tremor during climbing fiber stimulation at frequencies above the ones reported in this study. However, due to the risk of tissue damage around the stimulation electrode, such stimulation frequencies were not tested further.

## Discussion

### Results summary

Our results show that stimulating climbing fibers at 0.5–3 Hz induces a frequency-dependent, graded suppression of Purkinje cell activity. Climbing fiber stimulation at ≥4 Hz completely silences Purkinje cells and causes an abolition of conditioned blink responses, which are known to depend on the cerebellum. Given that these results were obtained in decerebrated, unanesthetized ferrets, we can exclude the possibility that the results were due to or influenced by anesthesia that has previously been shown to affect the cerebellum (Schonewille et al., 2006; Bengtsson and Jörntell, 2007).

### Relationship between climbing fiber stimulation and Purkinje cell activity

Prior studies have explored how olivary input shapes Purkinje cell activity. These studies have provided ample evidence, from several species, that lesioning or cooling the inferior olive increases Purkinje cell firing, whereas stimulating the climbing fibers suppresses or silences the Purkinje cells (Colin et al., 1980; Rawson and Tilokskulchai, 1981; Montarolo et al., 1982; Demer et al., 1985; Savio and Tempia, 1985; Cerminara and Rawson, 2004). However, these reports mainly show that higher stimulation frequencies induce stronger suppression of Purkinje cell activity and then report the threshold for completely silencing Purkinje cells. Moreover, the reported estimates have ranged between 2 Hz (Colin et al., 1980) and 8–10 Hz (Rawson and Tilokskulchai, 1981). Possible reasons for this variance are that the data come from different species and that different types of anesthesia were used. Our study thus adds to the existing body of evidence by providing a detailed description of the gradual suppression of Purkinje cell activity induced by small incremental increases in the frequency of climbing fiber stimulation, in an unanesthetized animal. Overall, the relation between climbing fiber stimulation and simple spike activity conforms to observations made in previous studies. For instance, Demer et al., (1985) found that stimulating at ≥4 Hz silences most, but not all, Purkinje cells. It is probably no coincidence that the inferior olive fires only at frequencies >5 Hz under artificial circumstances (Colin et al., 1980; Ekerot et al., 1987). In one cell, we did not see complete suppression even when stimulating climbing fibers at 10 Hz. This could indicate that we did not saturate the underlying mechanism responsible for the suppression. Alternatively, it means that some cells are not completely silenced by high-frequency climbing fiber stimulation, which would also be consistent with observations made by Demer et al., (1985).

How do climbing fibers suppress Purkinje cell activity? So far, two potentially complementary mechanisms have been proposed. First, climbing fiber stimulation can suppress Purkinje cells through activating inhibitory interneurons in the cerebellar cortex, which then inhibit Purkinje cells (Bloedel and Roberts, 1971; Schwarz and Welsh, 2001; Badura et al., 2013). In line with this alternative, optogenetic stimulation of the inferior olive activates basket cells, which are powerful inhibitors of Purkinje cells (Mathews et al., 2012). One argument against this mechanism is that subthreshold stimulation of climbing fibers, which does not elicit complex spikes but probably activates interneurons near the recorded Purkinje cell, did not affect Purkinje cell activity. Other researchers have proposed that climbing fiber activity inhibits Purkinje cells directly (Rawson and Tilokskulchai, 1981), possibly through activation of small-conductance, calcium-activated potassium channels, which are found in Purkinje cells (Hosy et al., 2011). Activation of these on the Purkinje cells causes a suppression of the neuronal activity lasting up to a couple of hundred milliseconds. The fact that Purkinje cells are silenced completely when climbing fibers are stimulated at 4 Hz may indicate that these channels mediate the inhibition. It is also important to remember that the cerebellum consists of different zones (Voogd, 1969; Oscarsson and Iggo, 1973; Armstrong, 1974; Ekerot and Larson, 1979; Voogd and Glickstein, 1998), which operate according to somewhat distinct molecular mechanisms (Zhou et al., 2014; Cerminara et al., 2015). Thus, the climbing fiber inhibition of Purkinje cell activity might not act uniformly across the cerebellum. Indeed, these zonal differences could potentially explain the variance in the suppression caused by climbing fiber stimulation, which has been observed in different studies.

The finding that even relatively low stimulation frequencies have effects on Purkinje cell activity highlights the importance of the regulatory effect of climbing fiber input. In addition to the suppression of simple spike firing, we sometimes also observed a suppression of the spontaneous complex spike activity. This was not further investigated here, and we can only speculate on the mechanisms behind the observation. Thus, as the simple spike activity drops, there should be an increase in the spontaneous activity in the nuclear cells. Presumably, this is true for the projection cells as well as the inhibitory olivary projecting cells (De Zeeuw and Berrebi, 1995). Consequently, the activity in the inferior olive, as well as the complex spike frequency in Purkinje cells, should be reduced (Andersson and Hesslow, 1987a,b). Importantly, the Purkinje cell firing returned to normal levels when high-frequency stimulation was stopped, suggesting that the sustained stimulation did not cause any pathological change to the Purkinje cells. It has been hypothesized that there is an optimal range of background activity within which the parallel fiber input is integrated (Cerminara and Rawson, 2004). This would ensure that the signal-to-noise ratio is kept within a range that is optimal both for synaptic plasticity and for Purkinje cell control of activity in the cerebellar nuclei and, hence, the output from the cerebellum (Bengtsson and Hesslow, 2013).

While the inferior olive regulates Purkinje cell activity, the activity of the olive itself is influenced by activation history (Hansel and Linden, 2000) and by GABAergic input from the cerebellar nuclei (Hesslow, 1986; Andersson et al., 1988; De Zeeuw et al., 1988; Nelson et al., 1989; Lang et al., 1996). As stated above, activation of the climbing fibers is bound to have subsequent effects all through the cerebello-olivary circuit (Bengtsson et al., 2004; Bengtsson and Hesslow, 2006). Moreover, direct stimulation of climbing fibers, as in our case, is bound to activate many climbing fibers, resulting in synchronous activation of many Purkinje cells (Lang et al., 1999). This synchronous activation will activate or inhibit downstream targets like the inhibitory interneurons (Szapiro and Barbour, 2007) and the cerebellar nuclei (Llinás and Mühlethaler, 1988; Bengtsson et al., 2011; Person and Raman, 2012). As the level of synchrony was not measured here, we cannot exclude that the activation may have had exaggerated circuitry effects (Bengtsson et al., 2011). There is also ample evidence that the inferior olive, which fires in bursts of 1-6 spikes (Maruta et al., 2007; Mathy et al., 2009), can be regulated in a graded manner (Najafi and Medina, 2013; Rasmussen and Hesslow, 2014), and that the number of spikes in an olivary burst can influence subsequent learning (Rasmussen et al., 2013, 2015; Yang and Lisberger, 2014). In other words, even a small increase in olivary activity can potentially cause changes to all parts of the cerebellar network, including the olive itself.

### Behavior

When extended to behavior, sustained climbing fiber stimulation in the intertrial period has a strong impact on the expression of ECRs. Stimulation at frequencies that silenced Purkinje cell activity also caused an abolition of ECRs. Given that there is a tight link between Purkinje cell activity and eyelid movements (Hesslow, 1994b; Heiney et al., 2014; Halverson et al., 2015), the present results suggest that high-frequency climbing fiber stimulation abolishes ECRs by silencing Purkinje cells, which leads to increased activity in the cerebellar nuclei and thus cerebellar output. In other words, high-frequency climbing fiber stimulation disrupts the entire cerebellar network and, in extension, cerebellum-dependent behavior. The fast recovery of ECRs when stimulation was stopped is consistent with the behavioral effect of injecting harmaline in the olive. Such injections influence NMDA receptors (Du and Harvey, 1997), leading to high levels of climbing fiber activity, which in turn prevent motor learning, including the acquisition of ECRs (Türker and Miles, 1984; Welsh, 1998). Our results are also in agreement with other pharmacological studies reporting an abolition of ECRs after manipulation of the inhibition of the cerebellar nuclei (Yeo et al., 1986; Parker et al., 2009). Suppression of the cerebellar nuclei through the application of a GABA agonist prevents conditioned responses because the nuclei cannot trigger motor responses. If instead activity in the cerebellar nuclei is increased through the application of a GABA antagonist or, as in this study, through climbing fiber stimulation, conditioned responses are abolished because of sustained activation of eyelid muscles, which prevents further modulation of the blink response (Aksenov et al., 2004; Parker et al., 2009).

### Conclusion

The fact that high-frequency climbing fiber stimulation disrupts Purkinje cell activity as well as cerebellum-dependent blink responses emphasizes the importance of considering the cerebellar network as a whole. Cerebellar function relies on the interaction among all parts of the cerebellar network, and disrupting any part of the network can affect other parts of the network in ways that can be difficult to anticipate. These effects are particularly important to consider when using anesthesia or when lesioning parts of the cerebellum, because such interventions can cast the entire system off balance.

## Synthesis

The decision was a result of the Reviewing Editor Christian Hansel and the peer reviewers coming together and discussing their recommendations until a consensus was reached. A fact-based synthesis statement explaining their decision and outlining what is needed to prepare a revision is listed below. The following reviewers agreed to reveal their identity: Samuel Wang, John Welsh

Overall, this is an interesting set of experiments that explores the functional implications of repetitive climbing fiber activation on simple spike activity and conditioned eyeblink responses. It adds information regarding the interplay between complex and simple spike activities in Purkinje cells and describes a functional consequence when repetitive climbing fiber activity is pathologically sustained. In general, the data seem a bit underspecified / underanalyzed. The authors might want to provide additional analysis (as specified below) to further strengthen the paper. For example, this work potentially provides the transfer function between the climbing-fiber teaching signal and Purkinje cell simple-spike output. The authors are in a unique position to identify that function.

This manuscript explores the effect of climbing-fiber-tract activation on the simple-spike firing rate of Purkinje cells in decerebrated ferrets. Because of the various ways in which climbing fiber action may influence Purkinje cell simple spiking, this is an important interaction that may play a substantial role in governing the excitability and responsiveness of Purkinje cells, which provide the output of the cerebellar cortex to the cerebellar nuclei.

This set of experiments studied the effect of repetitive electrical activation of climbing fibers on simple spike activity of Purkinje cells and on the performance of conditioned eyeblink responses in decerebrate ferrets. The experiment demonstrates, and confirms, in a novel way the phenomenon that repetitive climbing fiber activation impairs conditioned responses. This has been observed previously using 10 Hz pharmacological activation of the IO with harmaline during classical eyeblink conditioning in rabbits as they tremored (Turker &amp; Miles 1984; Du &amp; Harvey 1997). The effect may have general importance because it generalizes to other forms of learning, such as in operant tongue conditioning in rats (Welsh 1998). The current paper resolves the frequency dependence, which is more highly controlled electrically, showing that approximately 3 Hz activation is a threshold at which CRs are abolished. As this frequency correlates with the stimulation frequency that suppresses Purkinje cell simple spikes, it may be concluded that a suppression of simple spikes may contribute to the performance impairment.

This is an interesting paper that demonstrates robust effects. The recordings appear to be of high quality. Below are some comments that I would suggest be addressed by the authors - a mix of major and minor in order of the text.

1. Introduction, line 43: replace "peripheral input" with "input about the periphery" or simply "input." Except in rare instances, Purkinje cells do not receive peripheral input.

2. line 66. That IO output is modulated by feedback from the DCN was shown by Lang et al 1996, and should be cited and considered in the discussion.

3. line 124. Fig. 1B does not show the ISI distribution, as stated. Please show the "arechetypal interspike interval distributions."

4. line 167. Here, the interesting observation is made that a tremor corrupted the recorded EMG signal. In this regard it could be pointed out that repetitive climbing fiber stimulation is similar to harmaline, both in producing tremor and suppressing conditioned eyeblink responses. An EMG recording of the tremor may be of interest, as well as the implications of this for the last point below (point 11).

5. line 174. The number of Purkinje cells recorded is somewhat low (n=5). In the absence of collecting more Purkinje cells (requiring more decerebrations which may not be necessary), it would be helpful to show a more complete description of those cells with regard to their climbing fiber responses. In particular, it would be helpful to show the details of the CFRs triggered by periocular stimulation as well as interspike interval distributions.

6. line 214. It does not appear that the calculation of the contribution of repetitive climbing fiber activation on simple spike suppression considers the climbing fiber reflex (Eccles Llinas Sasaki J Physiol 1966, and other publications). This should be taken into account, especially given the distributed nature of the reflex response due to electrotonic coupling. This is relevant for point 10.

7. paragraph beginning line 243. No reference to Figures 3A and B.

8. lines 249, 255. Reference is made to Figure 3E, which does not exist.

9. General: reference is made often that repetitive climbing fiber stimulation >3 Hz "silences" Purkinje cells, but data are presented for simple spikes only. Are complex spikes also silenced during the acute period following repetitive icp stimulation? If so, what are the implications for Purkinje cell health, especially given the large Ca flux triggered by climbing fibers?

10. paragraph beginning line 290. That inhibitory interneurons contribute to post CS simple-spike pauses was suggested first by Bloedel &amp; Roberts (1971) and demonstrated to be due to their recruitment by climbing fiber synchrony (Schwarz &amp; Welsh 2001). In addition, calcium-dependent potassium conductances contribute to afterhyperpolarization effects and the complex spike pause (Hosy et al., J. Physiol., 2011). Proper scholarship suggests adding those references as well as considering the consequence of icp stimulation in producing highly synchronized ensemble complex spike activity.

Moreover, the authors should add references (and discuss) of data showing that the complex spike waveform itself can be modified depending on prior activation history (Hansel &amp; Linden 2000) and the number of climbing fiber spikes (Mathy et al, Neuron 62, 2009), respectively. These findings are important in the context of this manuscript as they indicate that the influence of the complex spike on simple spike firing is not constant.

11. line 325. Final sentence: in the very last sentence of the manuscript a major finding is stated for the first time that the "silencing of Purkinje cells" abolishes CRs due to sustained eyelid closure. This has strong implications for the manuscript and should be presented earlier, explored further, and figures presented. If true, then the suppression of CRs may have less to do with alterations in the expression of plasticity in Purkinje cells, than with motor effects that prevent CR expression. This point should be clarified. Did repetitive icp stimulation produce tonic activation of orbicularis oculi and what was the frequency dependence?

12. The work has the potential to be of use in understanding the olivocerebellar loop. However, the authors have not extracted nearly the value they could get out of the data set. Some fairly simple analyses could provide valuable reference values for future investigators - in some sense, a transfer function between CF input and SS output. I encourage the authors to do those analyses. In addition, add some clarifications for people who are not experts in in vivo cerebellar physiology.

From Figure 2c, it appears that steps up in frequency lead to decreases in firing rate within a few seconds. It would be helpful to plot the moment-to-moment change in firing rate following a step, for instance by plotting, as a function of time, 1/ISI averaged over a small number of spikes. This could be done for individual animals and/or averaged across animals. Show this for each frequency, and calculate a t_1/2 for change in simple-spike firing rate. Finally, plot t_1/2 as a function of frequency. This would be interpretable as the rate of accumulation of whatever factor X is responsible for simple-spike adaptation.

13. The example in Figure 2c also shows a case in which simple-spike firing rate recovers slowly, over tens of seconds. This is a bit deceptive since the neuron is totally silenced. Still, some plot of the recovery time course (calculated in a similar same way as above) would be informative. I see that Exp 4 (Figure 2b) was not totally silenced at 10 Hz, so that might be useful since factor X might not have saturated the silencing mechanism.

14. Stimulation of the climbing-fiber pathway using an electrode has the notable characteristic of activating many climbing fibers at once. This must be spelled out for readers in the Introduction and DIscussion. Since complex spike synchrony is known to activate several downstream targets in a special way (basket cells, see Szpara and Barbour) and deep nuclear cells (Person and Raman; also Schneider et al; also see the old Mühlethaler work), this approach adds a notable nonlinearity.

15. Broadly, the Discussion would be helped by a listing of possible mechanisms. I recognize that the authors' conservatism might lead them to be modest in this domain. But I think it would be educational for readers to understand the possibilities. I encourage the authors to do a good job of reviewing the possibilities in 1-2 well-done paragraphs. The possibilities will be constrained a bit by the analysis that I suggest in comments 1 and 2 above. Possibilities include co-activation and second-messenger accumulation in various cells. Immediately coming to mind are basket cells (Szpara and Barbour), calcium accumulation and therefore activation of calcium-activated conductances in Purkinje cells, and a similar adaptive process in deep nuclear neurons. There is also feedback via olivonuclear loops.

Overall, this is an interesting set of experiments that explores the functional implications of repetitive climbing fiber activation on simple spike activity and conditioned eyeblink responses. As such it could add important information to the literature once the issues described above are addressed.

Minor comment: Overall, the manuscript is clear. However, there are some subtle language errors, for instance misuse of the word "disability" in the abstract. Also, I detect some misplaced commas, subject-verb number disagreements, and compound sentences that could be simplified. I wonder if the authors could please ask for a close reading from some colleague who is fussy about English-language writing.

## Author Response

Overall, this is an interesting set of experiments that explores the functional implications of repetitive climbing fiber activation on simple spike activity and conditioned eyeblink responses. It adds information regarding the interplay between complex and simple spike activities in Purkinje cells and describes a functional consequence when repetitive climbing fiber activity is pathologically sustained. In general, the data seem a bit underspecified / underanalyzed. The authors might want to provide additional analysis (as specified below) to further strengthen the paper. For example, this work potentially provides the transfer function between the climbing-fiber teaching signal and Purkinje cell simple-spike output. The authors are in a unique position to identify that function.

Authors: We have added a scatterplot with a best-fit function depicting the relation between climbing fiber stimulation frequency and changes in simple spike firing (see panel C in Figure 2).

This manuscript explores the effect of climbing-fiber-tract activation on the simple-spike firing rate of Purkinje cells in decerebrated ferrets. Because of the various ways in which climbing fiber action may influence Purkinje cell simple spiking, this is an important interaction that may play a substantial role in governing the excitability and responsiveness of Purkinje cells, which provide the output of the cerebellar cortex to the cerebellar nuclei.

This set of experiments studied the effect of repetitive electrical activation of climbing fibers on simple spike activity of Purkinje cells and on the performance of conditioned eyeblink responses in decerebrate ferrets. The experiment demonstrates, and confirms, in a novel way the phenomenon that repetitive climbing fiber activation impairs conditioned responses. This has been observed previously using 10 Hz pharmacological activation of the IO with harmaline during classical eyeblink conditioning in rabbits as they tremored (Turker & Miles 1984; Du & Harvey 1997). The effect may have general importance because it generalizes to other forms of learning, such as in operant tongue conditioning in rats (Welsh 1998). The current paper resolves the frequency dependence, which is more highly controlled electrically, showing that approximately 3 Hz activation is a threshold at which CRs are abolished. As this frequency correlates with the stimulation frequency that suppresses Purkinje cell simple spikes, it may be concluded that a suppression of simple spikes may contribute to the performance impairment.

This is an interesting paper that demonstrates robust effects. The recordings appear to be of high quality. Below are some comments that I would suggest be addressed by the authors - a mix of major and minor in order of the text.

Authors: Thank you for the kind words, and for spotting the omission of some relevant papers. We now briefly mention the studies you bring up in the end of the discussion.

Checklist

1. Introduction, line 43: replace “peripheral input” with “input about the periphery” or simply “input.” Except in rare instances, Purkinje cells do not receive peripheral input.

Authors: We agree with the reviewer and have replaced peripheral input with just “input”

2. line 66. That IO output is modulated by feedback from the DCN was shown by Lang et al 1996, and should be cited and considered in the discussion.

Authors: We have added a paragraph in which we describe how activity of the inferior olive is regulated by the cerebellar nuclei, which in turn is regulated by Purkinje cell activity, thus forming an interdependent loop. See last paragraph in the introduction as well as the last paragraph in the electrophysiology part of the discussion section.

3. line 124. Fig. 1B does not show the ISI distribution, as stated. Please show the “arechetypal interspike interval distributions.”

Authors: We have added a figure illustrating the interspike interval distribution for a single cell as well as the interspike interval distribution for all cells and for each stimulation frequency. See figure 3.

4. line 167. Here, the interesting observation is made that a tremor corrupted the recorded EMG signal. In this regard it could be pointed out that repetitive climbing fiber stimulation is similar to harmaline, both in producing tremor and suppressing conditioned eyeblink responses. An EMG recording of the tremor may be of interest, as well as the implications of this for the last point below (point 11).

Authors: As we now state in the manuscript we did sometimes observe a tremor following repetitive climbing fiber stimulation, however, we did not examine this further and due to the risk of tissue damage around the stimulation electrode we curarized the animal whenever the tremor began.

5. line 174. The number of Purkinje cells recorded is somewhat low (n=5). In the absence of collecting more Purkinje cells (requiring more decerebrations which may not be necessary), it would be helpful to show a more complete description of those cells with regard to their climbing fiber responses. In particular, it would be helpful to show the details of the CFRs triggered by periocular stimulation as well as interspike interval distributions.

Authors: We have added a figure showing interspike interval distributions (see comment above). Regarding the CFRs elicited by periorbital stimulation, the five cells we recorded were all found near the area on the cerebellar cortex in which periocular stimulation generated the largest field potential. When the cells were found we also verified (visually) that periocular stimulation elicited a complex spike. All this is mentioned in the manuscript and it is standard procedure in our lab. However, we do not always save the traces from the periocular stimulation since they usually do not differ much in their appearance. Yet we are aware that there may be differences in the appearance of complex spikes elicited by climbing fiber stimulation and periocular stimulation. In these five cells there is however unfortunately not enough data to examine this in any meaningful way. We only have periocular traces from two of the five cells included in this analysis. An example from one of the cells is shown in Figure 1 in the paper.

6. line 214. It does not appear that the calculation of the contribution of repetitive climbing fiber activation on simple spike suppression considers the climbing fiber reflex (Eccles Llinas Sasaki J Physiol 1966, and other publications). This should be taken into account, especially given the distributed nature of the reflex response due to electrotonic coupling. This is relevant for point 10.

Authors: We now briefly discuss the climbing fiber reflex, stating that due to antidromic activation of the inferior olive, the climbing fiber activity may actually exceed the stimulation frequency. However, as we also state, we always adjusted the stimulation intensity to minimize the occurrence of extra complex spikes.

7. paragraph beginning line 243. No reference to Figures 3A and B.

Authors: We have added a sentence in the main text that briefly describes the content in these figures

8. lines 249, 255. Reference is made to Figure 3E, which does not exist.

Authors: Corrected

9. General: reference is made often that repetitive climbing fiber stimulation >3 Hz “silences” Purkinje cells, but data are presented for simple spikes only. Are complex spikes also silenced during the acute period following repetitive icp stimulation? If so, what are the implications for Purkinje cell health, especially given the large Ca flux triggered by climbing fibers?

Authors: Typically, the climbing fiber stimulation silences both simple spikes and spontaneous complex spikes, which is an interesting observation that we plan to examine more closely in a follow up paper. The fact that Purkinje cell activity returns to normal when climbing fiber stimulation is halted, suggests that the Purkinje cells were not damaged by the stimulation. This is now discussed in more depth in the manuscript.

10. paragraph beginning line 290. That inhibitory interneurons contribute to post CS simple-spike pauses was suggested first by Bloedel & Roberts (1971) and demonstrated to be due to their recruitment by climbing fiber synchrony (Schwarz & Welsh 2001). In addition, calcium-dependent potassium conductances contribute to afterhyperpolarization effects and the complex spike pause (Hosy et al., J. Physiol., 2011). Proper scholarship suggests adding those references as well as considering the consequence of icp stimulation in producing highly synchronized ensemble complex spike activity. Moreover, the authors should add references (and discuss) of data showing that the complex spike waveform itself can be modified depending on prior activation history (Hansel & Linden 2000) and the number of climbing fiber spikes (Mathy et al, Neuron 62, 2009), respectively. These findings are important in the context of this manuscript as they indicate that the influence of the complex spike on simple spike firing is not constant.

Authors: We expanded the discussion of possible mechanisms responsible for the silencing of the Purkinje cells and added the proper references. We have written about the potential role of the climbing fiber reflex in generating extra complex spikes and the implications of this for our data. We have also written a new paragraph about the regulation of the inferior olive, in which we discuss how the cerebellar nuclei regulates activity in the inferior olive and how the olive can also fire in a graded manner, with a variable number of spikes in each burst.

11. line 325. Final sentence: in the very last sentence of the manuscript a major finding is stated for the first time that the “silencing of Purkinje cells” abolishes CRs due to sustained eyelid closure. This has strong implications for the manuscript and should be presented earlier, explored further, and figures presented. If true, then the suppression of CRs may have less to do with alterations in the expression of plasticity in Purkinje cells, than with motor effects that prevent CR expression. This point should be clarified. Did repetitive icp stimulation produce tonic activation of orbicularis oculi and what was the frequency dependence?

Authors: This subject has been further elaborated on in the text. Briefly, silencing Purkinje cells leads to a cessation of nuclear inhibition, which in turn should cause increased activation of the orbicularis oculi muscles, leading to eyelid closure.

12. The work has the potential to be of use in understanding the olivocerebellar loop. However, the authors have not extracted nearly the value they could get out of the data set. Some fairly simple analyses could provide valuable reference values for future investigators - in some sense, a transfer function between CF input and SS output. I encourage the authors to do those analyses. In addition, add some clarifications for people who are not experts in in vivo cerebellar physiology.

From Figure 2c, it appears that steps up in frequency lead to decreases in firing rate within a few seconds. It would be helpful to plot the moment-to-moment change in firing rate following a step, for instance by plotting, as a function of time, 1/ISI averaged over a small number of spikes. This could be done for individual animals and/or averaged across animals. Show this for each frequency, and calculate a t_1/2 for change in simple-spike firing rate. Finally, plot t_1/2 as a function of frequency. This would be interpretable as the rate of accumulation of whatever factor X is responsible for simple-spike adaptation.

Authors: To illustrate the relation between climbing fiber stimulation and simple spike frequency we have added a scatterplot with these two variables and fit the data with an exponential model. In addition, we have added the ISI distributions for each stimulation frequency. Data to fully address this issue is however limited because at higher stimulation frequencies the cells were silent when stimulation began. Still we thank the reviewer for the encouragement to study this point further. Further analysis of the olivo-cerebellar effect will appear in a follow up paper.

13. The example in Figure 2c also shows a case in which simple-spike firing rate recovers slowly, over tens of seconds. This is a bit deceptive since the neuron is totally silenced. Still, some plot of the recovery time course (calculated in a similar same way as above) would be informative. I see that Exp 4 (Figure 2b) was not totally silenced at 10 Hz, so that might be useful since factor X might not have saturated the silencing mechanism.

Authors: Given that we have not pinpointed the suppression mechanism we can only speculate regarding the speed of recovery. We therefore hesitate to speculate about the speed of the recovery.

14. Stimulation of the climbing-fiber pathway using an electrode has the notable characteristic of activating many climbing fibers at once. This must be spelled out for readers in the Introduction and DIscussion. Since complex spike synchrony is known to activate several downstream targets in a special way (basket cells, see Szpara and Barbour) and deep nuclear cells (Person and Raman; also Schneider et al; also see the old Mühlethaler work), this approach adds a notable nonlinearity.

Authors: We have added a paragraph about the effect of electrically activating climbing fibers on the cerebellar cortex and nuclei.

15. Broadly, the Discussion would be helped by a listing of possible mechanisms. I recognize that the authors’ conservatism might lead them to be modest in this domain. But I think it would be educational for readers to understand the possibilities. I encourage the authors to do a good job of reviewing the possibilities in 1-2 well-done paragraphs. The possibilities will be constrained a bit by the analysis that I suggest in comments 1 and 2 above. Possibilities include co-activation and second-messenger accumulation in various cells. Immediately coming to mind are basket cells (Szpara and Barbour), calcium accumulation and therefore activation of calcium-activated conductances in Purkinje cells, and a similar adaptive process in deep nuclear neurons. There is also feedback via olivonuclear loops.

Authors: We have expanded the discussion about possible mechanisms explaining how climbing fibers silence Purkinje cells. We have of course also added references to relevant papers (thank you for the suggestions).

Overall, this is an interesting set of experiments that explores the functional implications of repetitive climbing fiber activation on simple spike activity and conditioned eyeblink responses. As such it could add important information to the literature once the issues described above are addressed.

Authors: We appreciate the kind words.

Minor comment: Overall, the manuscript is clear. However, there are some subtle language errors, for instance misuse of the word “disability” in the abstract. Also, I detect some misplaced commas, subject-verb number disagreements, and compound sentences that could be simplified. I wonder if the authors could please ask for a close reading from some colleague who is fussy about English-language writing.

Authors: All authors have gone through the manuscript carefully to correct language errors and we have also asked an acquaintance who is a native English speaker to go through the manuscript.
